# From Bench to Bedside—Implementing the New ABC Approach for Atrial Fibrillation in an Emergency Department Setting

**DOI:** 10.3390/ijerph19084797

**Published:** 2022-04-15

**Authors:** Sophie Gupta, Martin Lutnik, Jan Niederdöckl, Sebastian Schnaubelt

**Affiliations:** 1 Department of Emergency Medicine, Medical University of Vienna, 1090 Wien, Austria; sophie.gupta@meduniwien.ac.at (S.G.); sebastian.schnaubelt@meduniwien.ac.at (S.S.); 2 Department of Clinical Pharmacology, Medical University of Vienna, 1090 Wien, Austria; martin.lutnik@meduniwien.ac.at

## 1. The Novel ABC Approach Meets Emergency Medicine

Atrial fibrillation (AF) is a globally evolving medical challenge with, currently, 4% prevalence in the European Union’s population [1]. The number of affected people is estimated to increase dramatically in the next centuries: up to every fifth middle-aged person is likely to suffer from AF in their lifetime [2]. AF is linked to serious complications: The risk of developing ischemic stroke is elevated five-fold and comorbidities, such as heart failure, may worsen drastically [3]. 

Recent guidelines by the European Society of Cardiology (ESC) present a new ABC (Atrial fibrillation Better Care) approach to generate a structured, multidisciplinary, and holistic management of AF patients in order to improve treatment. This pathway includes proper anticoagulation to minimize stroke risk, better symptom management due to rhythm or rate control, and optimization of cardiovascular health and comorbidities [4]. By implementing this ABC approach, mortality and morbidity could be reduced significantly compared to usual care, as the mAFA-II trial showed [5].

Care provided at emergency departments (ED) plays a crucial role as AF accounts for 0.5–1% of ED admissions and shows to be the most common ED dysrhythmia [6,7,8]. However, data on AF management, especially regarding acute therapy, are still inconsistent. Apart from immediate electrical cardioversion (eCV) of hemodynamically instable patients, there are major differences in the use of rate and rhythm control—the main treatment approaches for AF. There is still no definite recommendation for or against either approach in the ESC guidelines since the two strategies show no significant difference in outcome [4,7,9,10]. AF management at EDs has a big impact on outcome of these patients, as respective patients have a higher hospitalization rate (~28%) than patients admitted for any other reason than AF (~17%) [11]. Insights from our ED reveal an increased short-term mortality, even of non-critically ill but symptomatic AF patients in comparison to the general population [12].

Taking these data into account, it can be assumed that AF patients presenting to an ED represent a special population. Therefore, an effective and practical implementation of the ABC approach including individualized risk stratification becomes necessary and is likely to improve outcomes. The ESC AF 2020 guidelines [4] point out a number of evidence gaps, especially regarding specific subgroups, for instance critically ill patients. The ED’s perspective as a central and crucial part of AF management might hereby serve as a pivotal point in facilitating an optimized ABC approach in various special circumstances. 

## 2. Identifying a Suitable Individual Treatment Approach

As mentioned, current literature is hesitant to provide a general recommendation on rhythm vs. rate control, as rhythm control still shows no overall superiority. However, some indications for rhythm control which are applicable at an ED are given by the ESC [4]: Younger age, first episode, AF related symptoms, few comorbidities, tachycardia-mediated cardiomyopathy, and patient’s choice. In short, a shared decision-making process taking into account the present symptom burden and comorbidities seems reasonable. 

There are two options available for restoration of sinus rhythm: Electrical cardioversion (eCV) with conversion rates up to 90% at first shock [13] and medical CV (mCV) with success rates ranging from 31% to 90% depending on the used agent [4]. There is also the possibility of combining drug and shock therapy to facilitated cardioversion, leading to higher converting rates: Alegret et al. showed that amiodaron administered prior to eCV led to higher success rates in comparison to no pre-treatment (89% vs. 86%). [14] Generally, a CV in an ED setting was shown to be safe and effective with high conversion rates up to 97% [6,8]. An early attempt of rhythm control might possibly improve symptoms and quality of life (QoL) in ED patients with AF [15]. Stiell et al. [16] showed that an ED strategy of immediate treatment leading to restoration of sinus rhythm and following discharge is safe. Readmission rates and symptom burden of AF decreased. In an emergency medicine setting, AF onset times can be difficult to determine. Moreover, means of thrombus detection, which is indicated in non-anticoagulated patients with an onset of >48 h as the Blitz-AF trial points out, might be an obstacle in a fast and effective pathway to cardioversion [11]: Transesophageal echocardiography (TOE) is listed as the current “gold standard”. In addition to good sensitivity of TOE, there are also possible complications such as esophageal perforation, which are very rare but life-threatening [17]. Additionally, TOE needs well-trained personnel and disrupts daily routine as it binds resources in an environment of time- and personnel-related shortages. Therefore, TOE may be suboptimal in the described setting. Mosleh et al. [18] displayed cardiac computed tomography (CCT) as alternative, and described that when comparing TOE to CCT, CCT was non-inferior in LAA thrombus detection [19]. This suggests CCT to be an effective alternative due to its broad availability and rapid performance. Thus, implementing CCT in AF protocols may increase CV rates and decrease readmissions. Further research endeavors should urgently be undertaken on this topic. 

## 3. Optimal Resource Use

CV—both electrical and pharmacological—must be performed under constant monitoring of vital signs. Therefore, a well-trained team and efficient standard operating procedures (SOP) are required to increase efficiency and reduce the impact of the procedure on resource usage. Bellew et al. [20] describe a significant improvement of outcome by using a standardized AF management algorithm based on the ESC AF guidelines, conducted at an ED. This algorithm includes evaluation of patients by stroke risk stratification and a defined pathway of agents and dose regimens, as well as management strategies (rate versus rhythm control), followed by an observation period and follow up. After deploying this algorithm, hospital admissions dropped without an increase of adverse events. Furthermore, Pluymaekers et al. reported that a wait-and-watch strategy in hemodynamically stable and rate-controlled patients with recent onset AF at an ER was not inferior to an early CV, with the endpoint being sinus rhythm after four weeks. In this trial, 69% of patients converted spontaneously within 48 h during or after initial rate control treatment. However, as Pluymaekers et al. pointed out, a possible disadvantage of a wait-and-watch strategy might be a prolonged symptom burden and negative effect on disease progression [21]. Therefore, a crucial part of an ED AF strategy should be the identification of patients with a high probability of spontaneous conversion, leading to fewer interventions and—naturally—fewer associated complications. In this regard, the so-called ReSinus Score [22] might be a sufficient and useful tool to evaluate spontaneous conversion by merging biomarkers and clinical characteristics.

## 4. Risk Assessment

General risk assessment of AF patients should be an important task at an ED. Particular attention should be paid to the increased stroke risk in AF patients. If not contraindicated, early and sufficient anticoagulation (AC) is highly recommended by the ABC approach [4]. The HERMES-AF trial [23] shows that AF patients presenting to an ER often have various comorbidities and are, therefore, at high risk of stroke. However, Kea et al. [24] point out that many of those patients are still eligible discharged without a respective prescription. Therefore, a strict evaluation of stroke risk based on the CHA2DS2-VASc Score is required and must find its place in ED management algorithms.

Biomarkers, such as D-dimer, high-sensitive cardiac troponins, or brain natriuretic peptide (BNP), provide additional means of risk stratification regarding new onset AF, as well as disease characterization and progression [25]. The ABC score evaluates stroke and bleeding risk by using 13 biomarkers [26]. A previous study at our site shows that elevated troponin and BNP levels predict a higher mid-term mortality in symptomatic AF patients [27]. In summary, a recommendation for a more extensive use—and further research into biomarkers in ED AF patients can be deducted.

## 5. Patient Education

Another important milestone of the ABC approach is patient education [4]. An understanding of the disease is crucial for compliance, and it has been shown that lacking general knowledge of the own AF illness and especially the importance of AC lead to unfavorable outcomes: For example, Hernandez Madrid et al. showed that a lower level of education regarding AC led to higher bleeding events and INR peaks exceeding the upper limit. An explanation of AF and heart rhythm disorders, complications, such as stroke, self-care, and possible precautions conducted at discharge from the ED lowers hospital readmissions and AF-related complications [28]. Several paths open up for education and training, for example providing information orally by trained nurses or physicians like Fuenzalida et al. showed [28], or in form of easily understandable flyers like Brand et al. [29] reported. Flyers may be an efficient option due to limited ED resources.

## 6. Conclusions

Implementing the new ABC approach for AF in the setting of an ED seems necessary and useful since its simplicity and potential benefits on outcomes. However, further SOPs and treatment protocols are essential to achieve optimized management, especially when assessing various ED subpopulations such as critically ill individuals. SOPs provide a constant level of quality and help so no important information is missed during stressful clinical routine. Patients’ individual risks and needs should be evaluated by scores, so that sufficient AC can be ensured. To further improve risk stratification, extended patient evaluation including laboratory markers should be conducted. Investigations that can be partially outsourced (e.g., laboratory, CCT) can aid in conserving ED resources. Better symptom care and improvement of QoL could be implemented by an individualized and early targeted treatment, focusing on CV for eligible patients. This could be enforced by CCT if TOE is not practical. Finally, patient education and a patient-centered approach can facilitate a holistic AF management. As Pokorney et al. have already pointed out: “Is it really as simple as ABC?” [30].

## References

[1] Ball J., Carrington M.J., McMurray J.J.V., Stewart S. (2013). Atrial fibrillation: Profile and burden of an evolving epidemic in the 21st century. Int. J. Cardiol..

[2] Zoni-Berisso M., Lercari F., Carazza T., Domenicucci S. (2014). Epidemiology of atrial fibrillation: European perspective. Clin. Epidemiol..

[3] Zulkifly H., Lip G.Y.H., Lane D.A. (2018). Epidemiology of atrial fibrillation. Int. J. Clin. Pract..

[4] Hindricks G., Potpara T., Dagres N., Arbelo E., Bax J.J., Blomström-Lundqvist C., Boriani G., Castella M., Dan G.A., Dilaveris P.E. (2020). 2020 ESC Guidelines for the diagnosis and management of atrial fibrillation developed in collaboration with the European Association of Cardio-Thoracic Surgery (EACTS). Eur. Heart J..

[5] Guo Y., Lane D.A., Wang L., Zhang H., Wang H., Zhang W., Wen J., Xing Y., Wu F., Xia Y. (2020). Mobile Health Technology to Improve Care for Patients With Atrial Fibrillation. J. Am. Coll. Cardiol..

[6] Atzema C.L., Austin P.C., Miller E., Chong A.S., Yun L., Dorian P. (2013). A population-based description of atrial fibrillation in the emergency department, 2002 to 2010. Ann. Emerg. Med..

[7] Rogenstein C., Kelly A.-M., Mason S., Schneider S., Lang E., Clement C.M., Stiell I.G. (2012). An international view of how recent-onset atrial fibrillation is treated in the emergency department. Acad. Emerg. Med..

[8] Stiell I.G., Sivilotti M.L.A., Taljaard M., Birnie D., Vadeboncoeur A., Hohl C.M., Mcrae A., Rowe B.H., Brison R.J., Thiruganasambandamoorthy V. (2020). Electrical versus pharmacological cardioversion for emergency department patients with acute atrial fibrillation (RAFF2): A partial factorial randomised trial. Lancet.

[9] Hamilton A., Clark D., Gray A., Cragg A., Grubb N. (2015). Emergency Medicine Research Group, Edinburgh (EMERGE). The epidemiology and management of recent-onset atrial fibrillation and flutter presenting to the Emergency Department. Eur. J. Emerg. Med..

[10] Atzema C.L., Singh S.M. (2018). Acute Management of Atrial Fibrillation: From Emergency Department to Cardiac Care Unit. Cardiol. Clin..

[11] Gulizia M.M., Cemin R., Colivicchi F., De Luca L., Di Lenarda A., Boriani G., Di Pasquale G., Nardi F., Scherillo M., Lucci D. (2019). Management of atrial fibrillation in the emergency room and in the cardiology ward: The BLITZ AF study. EP Europace.

[12] Niederdöckl J., Schwameis M., Herkner H., Domanovits H. (2021). Excess short-term mortality in noncritical patients with atrial fibrillation presenting to the emergency department. Wien. Klin. Wochenschr..

[13] Burton J.H., Vinson D.R., Drummond K., Strout T.D., Thode H.C., McInturff J.J. (2004). Electrical cardioversion of emergency department patients with atrial fibrillation. Ann. Emerg. Med..

[14] Alegret J.M., Viñolas X., Tajes H., Valdovinos P., Palomares R., Arias M.A., Bazán V. (2020). Utility of Amiodarone Pre-Treatment as a Facilitator of the Acute Success of Electrical Cardioversion in Persistent Atrial Fibrillation. Cardiovasc. Drugs Ther..

[15] Penttilä T., Mäkynen H., Hartikainen J., Hyppölä H., Lauri T., Lehto M., Lund J., Raatikainen M.J. (2017). Antiarrhythmic drug therapy among patients presenting to emergency department with symptomatic atrial fibrillation—A prospective nationwide cohort. Scand. J. Trauma Resusc. Emerg. Med..

[16] Stiell I.G., Clement C.M., Rowe B.H., Brison R.J., Wyse D.G., Birnie D., Dorian P., Lang E., Perry J.J., Borgundvaag B. (2017). Outcomes for Emergency Department Patients with Recent-Onset Atrial Fibrillation and Flutter Treated in Canadian Hospitals. Ann. Emerg. Med..

[17] Chan K.L., Cohen G.I., Sochowski R.A., Baird M.G. (1991). Complications of transesophageal echocardiography in ambulatory adult patients: Analysis of 1500 consecutive examinations. J. Am. Soc. Echocardiogr..

[18] Mosleh W., Sheikh A., Said Z., Ahmed M.A.-A., Gadde S., Shah T., Wilson M.F., Beck H., Kim C., Sharma U.C. (2018). The use of cardiac-CT alone to exclude left atrial thrombus before atrial fibrillation ablation: Efficiency, safety, and cost analysis. Pacing Clin. Electrophysiol. PACE.

[19] Spagnolo P., Giglio M., Di Marco D., Cannaò P.M., Agricola E., Della Bella P.E., Monti C.B., Sardanelli F. (2021). Diagnosis of left atrial appendage thrombus in patients with atrial fibrillation: Delayed contrast-enhanced cardiac CT. Eur. Radiol..

[20] Bellew S.D., Bremer M.L., Kopecky S.L., Lohse C.M., Munger T.M., Robelia P.M., Smars P.A. (2016). Impact of an Emergency Department Observation Unit Management Algorithm for Atrial Fibrillation. J. Am. Heart Assoc..

[21] Pluymaekers N.A.H.A., Dudink E.A.M.P., Luermans J.G.L.M., Meeder J.G., Lenderink T., Widdershoven J., Bucx J.J., Rienstra M., Kamp O., Van Opstal J.M. (2019). Early or Delayed Cardioversion in Recent-Onset Atrial Fibrillation. N. Engl. J. Med..

[22] Niederdöckl J., Simon A., Cacioppo F., Buchtele N., Merrelaar A., Schütz N., Schnaubelt S., Spiel A., Roth D.O., Schörgenhofer C. (2021). Predicting spontaneous conversion to sinus rhythm in symptomatic atrial fibrillation: The ReSinus score. Eur. J. Intern. Med..

[23] Coll-Vinent B., Martín A., Malagón F., Suero C., Sánchez J., Varona M., Cancio M., Sánchez S., Montull E., del Arco C. (2015). Stroke prophylaxis in atrial fibrillation: Searching for management improvement opportunities in the emergency department: The HERMES-AF study. Ann. Emerg. Med..

[24] Kea B., Waites B.T., Lin A., Raitt M., Vinson D.R., Ari N., Welle L., Sill A., Button D., Sun B.C. (2020). Practice Gap in Atrial Fibrillation Oral Anticoagulation Prescribing at Emergency Department Home Discharge. West. J. Emerg. Med..

[25] Noubiap J.J., Sanders P., Nattel S., Lau D.H. (2021). Biomarkers in Atrial Fibrillation. Card. Electrophysiol. Clin..

[26] Berg D.D., Ruff C.T., Morrow D.A. (2021). Biomarkers for Risk Assessment in Atrial Fibrillation. Clin. Chem..

[27] Niederdöckl J., Simon A., Schnaubelt S., Schuetz N., Laggner R., Sulzgruber P., Spiel A.O., Herkner H., Laggner A.N., Domanovits H. (2019). Cardiac biomarkers predict mortality in emergency patients presenting with atrial fibrillation. Heart.

[28] Fuenzalida C., Hernández G., Ferro I., Siches C., Ambrós À., Coll-Vinent B. (2017). Long-term benefits of education by emergency care nurses at discharge of patients with atrial fibrillation. Int. Emerg. Nurs..

[29] Brand A., Stangl V. Comics zur Verbesserung der Patientenaufklärung. https://herz.charite.de/forschung/klinische_forschung/comics_zur_verbesserung_der_patientenaufklaerung/.

[30] Pokorney S.D., Piccini J.P. (2021). Treatment of atrial fibrillation: Is it as easy as A-B-C?. EP Eur..

